# Early-Life Circumstances, Health Behavior Profiles, and Later-Life Health in Great Britain

**DOI:** 10.1177/0898264320981233

**Published:** 2020-12-19

**Authors:** Thijs van den Broek

**Affiliations:** 1 6984 Erasmus University Rotterdam, Rotterdam, the Netherlands

**Keywords:** life course, allostatic load, health behavior, biomarkers, health inequalities

## Abstract

**Objectives:** Drawing on UK Household Longitudinal Study data, this study assessed a pathway from early-life disadvantage to suboptimal later-life health via health behavior. **Methods:** Latent class analysis was used to identify distinct smoking, nutrition, alcohol, and physical activity health behavior profiles. Mediation analyses were performed to assess indirect effects of early-life disadvantage via health behavior on allostatic load, an objective measure of physiological wear and tear. **Results:** Four health behavior profiles were identified: (1) *broadly healthy and high alcohol consumption*, (2) *low smoking and alcohol consumption*, *healthy nutrition, and physically inactive*, (3) *broadly unhealthy and low alcohol consumption,* and (4) *broadly moderately unhealthy and high alcohol consumption*. Having grown up in a higher socioeconomic position family was associated with lower later-life allostatic load. This was partly attributable to health behavioral differences. **Discussion:** Growing up under disadvantageous socioeconomic circumstances may initiate a chain of risk by predisposing people to health behavior profiles associated with poorer later-life health.

## Introduction

Poor childhood conditions can be seen as “the launch pad for a lifetime of health problems” (Raphael, 2011, p. 24). Research consistently shows that growing up under disadvantageous circumstances, such as in poor financial conditions or not living with both parents, is associated with poor later-life health (Gilman et al., 2003; Gruenewald et al., 2012; Kumari et al., 2013; Latham-Mintus & Aman, 2019; Pakpahan et al., 2017). Such experiences may have an immediate impact on the nervous, endocrine, and immune systems that remains apparent in adulthood and later life (Danese & McEwen, 2012). Life course epidemiologists have argued that adversity experienced in childhood may also be harmful for later-life health in an indirect way. Poor childhood circumstances may initiate a *chain of risk*, that is, they may increase the risk of subsequent experiences or events that are detrimental for health (Kuh et al., 2003).

Consistent with the idea of chains of risk, research shows that children who grew up in poor economic circumstances tend to have a lower socioeconomic position as adults (Barboza Solís et al., 2016; Jenkins & Siedler, 2007), which is, in turn, associated with poorer adult health (Barboza Solís et al., 2016; Präg & Richards, 2019; Robert & House, 1996; Whitley et al., 2018). The intergenerational transmission of divorce is also well documented (Dronkers & Härkönen, 2008). People who experienced the divorce of their parents in childhood are moreover relatively likely to become parents at a young age (Quinlivan et al., 2004; Woodward et al., 2004). Divorce and early transitions to parenthood are both, in turn, known antecedents of poor later-life health (Grundy & Read, 2015; Lorenz et al., 2006; Rote, 2017; Sironi et al., 2020).

The current study’s focus is on a potential chain of risk via health lifestyles. Cockerham (2005) has argued that the disposition toward particular health lifestyles is the result of the interplay between *life chances* and *life choices*. Both life chances–the lifestyle options that are available to particular people–and life choices–the choices that people make from the options available–are, in part, shaped by structural variables, such as class circumstances and the so-called collectivities, that is, “collections of actors linked together through particular social relationships, such as kinship” (Cockerham, 2005, p. 59). Fewer lifestyle options for healthy living are available when growing up in poor economic conditions, for instance because healthy diets tend to be less affordable than unhealthy alternatives (Drewnowski, 2013) and because lack of money or access to transport are a particularly strong barrier to sports participation for members of families with a lower socioeconomic position (Chinn et al., 1999).

Health behavioral choices within the set of options that are available are also shaped by structural factors via socialization patterns and experiences (Cockerham, 2005). Growing up in poor economic conditions or in a single parent family may be detrimental for the acquisition of cultural capital, that is, the symbolic and informational resources, such as knowledge, values, and norms, that are acquired through social learning (Abel, 2008). People who grew up under such circumstances may thus have relatively poor opportunities for the social learning of healthy lifestyle behavior. Plausibly due to limited life opportunities and exposure to material hardship, people with a low socioeconomic position are relatively likely to view their health as something beyond their control, to think less of the future, and to think less about how they can stay healthy, all of which is, in turn, associated with unhealthy lifestyle choices (Wardle & Steptoe, 2003). Certain forms of unhealthy behavior, such as smoking or drinking, may provide persons with a short-term reduction of the stress associated with having grown up under disadvantageous circumstances, albeit at the expense of a broad range of longer-term health outcomes (McEwen & Stellar, 1993). Growing up in a disrupted family or under poor economic circumstances is furthermore associated with greater impulsivity (Peterson & Zill, 1986), which, in turn, is associated with unhealthy eating, drinking, and smoking behavior (Hofmann et al., 2008).

Adverse health effects of various forms of health behavior may offset or compound each other (Shaw & Agahi, 2012). Therefore, many scholars have recently adopted a holistic perspective on health behavior and considered various aspects of health behavior, such as smoking, drinking, diet, and physical activity, conjointly, rather than in isolation, to provide insights into which behavioral combinations should be prioritized for interventions (Burgard et al., 2020; Griffin et al., 2014; Saint Onge & Krueger, 2017; Shaw & Agahi, 2012). Although research in which multiple aspects of health behavior are combined into an index has provided valuable insights on the antecedents and later-life consequences of health behavior in general (e.g., Barboza Solís et al., 2016; Zaninotto et al., 2020), it should be noted that various forms of health behavior are only weakly associated (Newsom et al., 2005). Many people’s behavioral patterns are discordant, that is, neither uniformly healthy nor uniformly unhealthy (Saint Onge & Krueger, 2017). This suggests that health behavior is a multidimensional concept that cannot fully be captured with an index.

Acknowledging the multidimensional nature of health behavior, the current study uses latent class analysis to distinguish distinct health behavior profiles that vary on the so-called smoking, nutrition, alcohol, and physical activity (SNAP) behavioral dimensions among people aged 50–80 years in Great Britain. It also assesses how disadvantageous early-life circumstances, such as growing up under poor economic conditions or not with both parents, are associated with having particular health behavior profiles and the extent to which effects of disadvantageous early-life characteristics on later-life health is attributable to differences in health behavior profiles.

The outcome of interest is allostatic load, that is, the physiological wear and tear of the body due to repeated or chronic exposures to stressors (McEwen & Stellar, 1993). When people are confronted with stressful challenges, neural, neuroendocrine, and neuroendocrine–immune mechanisms are activated in response. Although beneficial in the short term, this response called allostasis–stability through change–comes with increased physiological wear and tear over time. This long-term damage is called allostatic load. McEwen (1998) has argued that health behavior should be regarded as part of the overall notion of allostasis. This is because suboptimal health behavior, for example, smoking or drinking, may help individuals to cope with stress and challenges in the short term, while being physiologically damaging in the long term (e.g., Barboza Solís et al., 2016; Gruenewald et al., 2012).

## Data and Methods

### Analytical Approach

Latent class analysis (LCA) (McCutcheon, 1987) will be used to identify distinct health behavior profiles that vary on multiple SNAP dimensions. Health behavior profiles are operationalized here as a latent categorical variable underlying response patterns on a range of survey questions covering different SNAP dimensions. Given that, LCA uses the expectation–maximization (EM) algorithm that may only produce a local rather than the global maximum of the log-likelihood function dependent on the initial parameter values chosen in the first iteration, all latent class models are estimated 250 times with different starting values. A model with two latent classes is first estimated and the number of classes is then increased until the addition of a latent class does not lead to a model fit improvement as indicated by the Bayesian information criterion (BIC) score (Schwarz, 1978). Given the current study’s considerable sample size (see next subsection), BIC is arguably the most appropriate criterion to detect the number of classes because it sufficiently penalizes model complexity to avoid overfitting (Dziak et al., 2020; Tein et al., 2013). The AIC score (Akaike, 1974) is a commonly used alternative criterion to determine the number of classes, but, although it may work well with small samples, it is marred by high overfitting rates in larger samples (Dziak et al., 2020).

After estimating the LCA model with the optimal number of classes, the posterior probabilities of class memberships for each class will be stored for every respondent. Multinomial logistic regression will subsequently be used to predict class membership, whereby uncertainty regarding class membership is taken into account through the use of weights inversely related to the class membership classification errors (Bolck et al., 2004).

Linear regression is used to predict allostatic load. In a first model, allostatic load is regressed on early-life characteristics and a range of contemporaneous controls. The health behavior profiles identified in the LCA are added in a second model. Weights inversely related to the membership classification errors are again used to take uncertainty regarding class membership into account (Bakk & Vermunt, 2016). Bootstrapping is used to estimate the indirect effect of early-life characteristics via health behavior profiles on allostatic load (Preacher & Hayes, 2008).

### Sample

Data are from the UK Household Longitudinal Study (UKHLS) (University of Essex, Institute for Social and Economic Research, NatCen Social Research, & Kantar Public, 2018, 2019). The UKHLS is a prospective, nationally representative study. Nurse visits took place in Wave 2, collected between 2010 and 2012. During these visits, physical measures, blood samples, and other health-related information were collected.

Analyses were restricted to 4700 people who participated in both Wave 1 and Wave 2, were aged between 50 and 80 years when Wave 2 data were collected, and had a valid blood sample analytical weight. 51 respondents were dropped because they reported that they did not live with at least one biological parent while growing up. This made it impossible to derive information on childhood socioeconomic circumstances. This exclusion procedure resulted in a final analytical sample of 4649 respondents.

Participation in the UKHLS nurse health assessment was selective. Most notably, no nurse visits were carried out in Northern Ireland. Furthermore, people with particular sociodemographic characteristics (e.g., people who were not married, lower educated, or not born in the UK) were underrepresented. The UKHLS team therefore prepared weights specially for the biomarker data to enable estimation samples to be representative of the general population of Great Britain (Benzeval et al., 2014). These supplied biomarker weights were used in this study.

### Measures

Given the analytical approach, three types of measures are distinguished: manifest items, explanatory variables, and the distal outcome. Manifest items are observed realizations of the underlying dimensions of the latent health behavior profiles. The distal outcome is a measure of later-life health predicted by one’s health behavior profile. Explanatory variables considered are background variables that are plausibly predictive of having a particular health behavior profile as well as of allostatic load.

### Manifest Items

Six smoking, nutrition, alcohol, and physical activity items were considered. With regard to smoking, current smokers, former smokers, and people who never smoked were distinguished. Two nutrition items were included: one capturing the frequency of fruit consumption and other capturing the frequency of vegetable consumption. Respondents were asked how often they consumed fruit and how often they consumed vegetables in a usual week, with response categories being never, 1–3 days per week, 4–6 days per week, or every day. The two bottom categories were combined into a new category for all respondents who consumed fruit, respectively vegetables, 0–3 days per week because very few respondents reported never eating any fruit or vegetables.

Respondents were also asked how often in the last week they consumed at least one alcoholic drink, and how many types of alcoholic drinks (beer or cider; alcohol shots, wine, and alcopops) they had on the day in the last week on which they drank the most. Alcohol consumption was subsequently converted to alcohol units, with one unit being equal to 10 ml of pure alcohol. Following the guidelines for low-risk drinking agreed upon by the UK chief medical officers (Department of Health and Social Care, 2016), respondents were categorized as nondrinkers, people with low-risk drinking behavior (≤14 units per week), or people with risky drinking behavior (>14 units per week).

Two physical activity items were considered. The question how many days of the past 4 weeks respondents had walked for at least 10 minutes continuously was used to capture the frequency of low-intensity physical activities (cf. Hughes & Kumari, 2017). A distinction was made between respondents who did so on not more than seven of the last 28 days, respondents who did so between 8 and 21 of the last 28 days, and respondents who did so on 22 or more of the last 28 days. A range of moderate-intensity sports were also considered, whereby a distinction was made between respondents who had done sports two times or less in the last year, respondents who had done sports more than twice in the last year, but less than weekly, and respondents who had done sports at least weekly.

### Distal Outcome

Allostatic load is a measure of the physiological wear and tear of the body due to repeated or chronic exposure to stressors. McEwen and Stellar (1993), who coined the concept, defined it as “the cost of chronic exposure to fluctuating or heightened neural or neuroendocrine response resulting from repeated or chronic environmental challenge that an individual reacts to as being particularly stressful” (p. 2093). Allostatic load is typically measured with an index of markers of various biological systems (for an overview of various operationalizations, see Johnson et al., 2017). Johnson et al. (2017) recently pointed out that there is no standard method of calculating an allostatic load index in the scholarly literature. What they deemed particularly problematic was the absence of hypothalamic–pituitary–adrenal (HPA) axis biomarkers in the operationalizations of allostatic load in approximately half of the studies they considered in their review. They argued that the absence of HPA-axis markers made the operationalization of allostatic load inconsistent with McEwen and Stellar’s conceptual definition in which the neuroendocrine response to stress was central. However, the UKHLS data used here did include an HPA-axis marker: dehydroepiandrosterone sulphate (DHEA-S). Dehydroepiandrosterone (DHEA) and its sulfate form DHEA-S are the most common steroid hormones in the body and their levels decline with age (Benzeval et al., 2014). In addition to DHEA-S, 12 other markers of cardiovascular (systolic blood pressure, diastolic blood pressure, and resting heart rate), metabolic (waist-to-height ratio, total cholesterol-to-HDL cholesterol ratio, HDL cholesterol, triglycerides, glycated hamoglobin (HbA1c), and insulin-like growth factor-1), kidney liver function (creatinine clearance rate), and immune response (C-reactive protein and fibrinogen) biological systems were included in the allostatic load measure.

Consistent with earlier work (Seeman et al., 2001), all 13 indicators were dichotomized based on quartiles in the weighted sex-specific distributions, whereby scores in the least favorable quartile were coded as one (See Supplementary Appendix A). The dichotomized items were summed into a scale ranging from 0 to 13, with higher scores indicating a poorer physiological condition. The scale’s internal consistency was acceptable (KR-20 = .59).

### Explanatory Variables

Early-life characteristics taken into account included whether or not respondents lived with both biological parents when they were 16 years old and whether or not they were born in the United Kingdom. Parental socioeconomic position when growing up was also taken into account. Respondents were asked to list the occupations of their parents when they were aged 14 years, and these occupations were subsequently coded according to the National Statistics Socioeconomic Classification. A distinction was made between parents with disadvantaged (“semi-routine and routine”), intermediate (“intermediate,” “small employers and own account,” and “lower supervisory and technical”), and advantaged (“management and professional”) socioeconomic positions. When the respondent reported that the parent was not working when the respondent was 14 years old, the parent was also coded as having low socioeconomic position. Where the socioeconomic positions of the father and the mother differed, the parental socioeconomic position was coded according to the parent with the highest socioeconomic position.

Early-life health was also considered. Respondents were asked if they were ever diagnosed with any of a range of diseases and health conditions, including diabetes, coronary heart disease, and clinical depression. Respondents who answered affirmatively were subsequently asked at what age they were told that they had this health condition. Respondents who were diagnosed with any of the listed health conditions at the age of 18 years or younger were coded as having health problems when growing up. Diseases most commonly mentioned as being diagnosed with when growing up were asthma and chronic bronchitis.

Contemporaneous explanatory variables taken into account are marital status, number of children, and educational attainment. A fourfold distinction was made by marital status: (i) currently in a marriage or civil union, (ii) divorced or separated, (iii) widowed, and (iv) never married. Given the nonlinear effects of fertility on health (Högnäs et al., 2017), number of children was coded as a categorical variable, with categories of 0, 1, 2, 3, and 4+ children (cf. Grundy et al., 2019; Sironi et al., 2020). Respondents’ educational attainment was coded according to their highest educational qualification. A distinction was made between low (GCSE [high school qualifications taken at age 16 years] or less), intermediate (at least A-level secondary education, but no university degree), and high (university degree) educational attainment. Age in years was also included as an explanatory variable. Sample characteristics are presented in Table 1.

**Table 1.** Sample Characteristics, Means, and Percentages.

	Women and Men (*n* = 4649)	Women (*n* = 2551)	Men (*n* = 2098)
Woman	55.0%		
Allostatic load	3.2	3.2	3.2
(*SD*)	(2.2)	(2.3)	(2.1)
Age	62.8	62.8	62.9
(*SD*)	(8.5)	(8.5)	(8.4)
Parental socioeconomic position			
Low	39.9%	39.0%	41.0%
Intermediate	36.3%	38.0%	34.2%
High	23.8%	23.0%	24.8%
Did not grow up with both parents	16.1%	15.7%	16.6%
Not born in the UK	7.4%	7.1%	7.9%
Diagnosed disease at the age of 18 years	5.3%	5.7%	4.9%
Marital status			
Married	64.0%	59.7%	69.3%
Divorced	16.6%	18.5%	14.3%
Widowed	10.7%	14.7%	5.8%
Never married	8.6%	7.0%	10.6%
Number of children			
No children	16.6%	14.3%	19.4%
1 child	12.6%	12.1%	13.3%
2 children	39.2%	40.2%	37.9%
3 children	20.0%	21.5%	18.1%
4 or more children	11.7%	11.9%	11.3%
Educational attainment			
Low	56.5%	62.8%	48.7%
Intermediate	27.1%	24.6%	30.2%
High	16.4%	12.6%	21.1%

*Notes*. Data are from Understanding Society–The UK Household Longitudinal Study; Data are weighted; Multiple imputation using chained equations used to deal with missing data.

### Missing Values

The sample included 1636 respondents (35.2%) with missing information on at least one variable of interest. Information on blood pressure (*n* = 711), drinking behavior (*n* = 551), HbA1c (*n* = 337), and C-reactive protein (*n* = 144) was missing most often. For all variables except the manifest items used to identify the latent categorical health behavior profiles, multiple imputation with chained equations were used to deal with missing information (Brand et al., 2019). The missing at random (MAR) assumption underlying this way of imputing missing information entails that any differences between the distributions of missing values and the distributions of observed values are to be explained by variables included in the imputation model. The findings from the substantive analyses on 20 imputed data sets were combined into a single set of results following Rubin’s rules (Little & Rubin, 1989), which take the variability in results between the imputed datasets into account. The iterative nature of the EM algorithm allows LCA-models to be estimated with missing observations on manifest health behavior items (Dempster et al., 1977), whereby MAR is also assumed.

## Results

### Four Health Behavior Profiles

**Figure 1.**
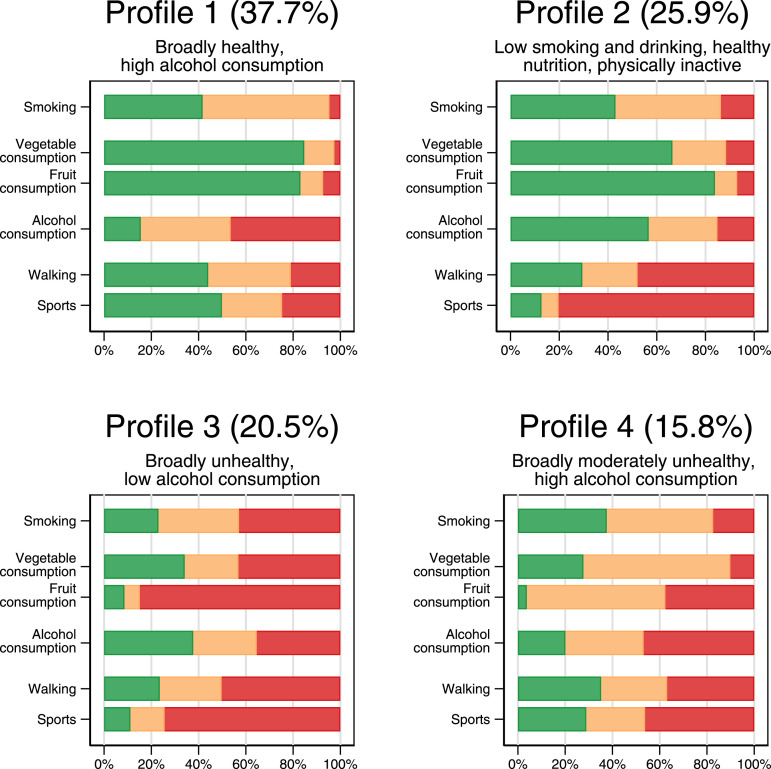
Class-conditional response probabilities.

A comparison of fit statistics indicated that a solution with four classes fitted optimally with our data (See Supplemental Appendix B). The BIC was lowest when a 4-class solution was chosen. The Akaike information criterion (AIC), which penalizes model complexity much more weakly, showed an “elbow” at the 4-class point, that is, there were only small further AIC declines with the addition of higher-order classes. Figure 1 provides an overview of the class conditional response probabilities on the considered manifest health behavior dimensions. The response probabilities displayed in green represent the class-conditional likelihood of being in the most favorable category in the particular behavioral dimension, respectively: never smoked, daily fruit consumption, daily vegetable consumption, nondrinker, walking for 10+ minutes continuously on 22 or more days out of the last 28 days, and at least weekly sports participation. In contrast, the response probabilities displayed in red respectively represent the class-conditional likelihood of being a current smoker, consuming fruit only 0–3 days a week, consuming vegetables only 0–3 days a week, drinking beyond sensible limits, walking for 10 minutes or more on not more than 7 days out of the last 28, and participating in sport fewer than three times a year.

The latent health behavior profile with the largest prevalence was characterized by a low probability of smoking and a high probability of frequent consumption of fruit and vegetables. Moreover, people with this profile were relatively likely to be physically active as indicated by a high probability of frequent walking and participation in sports. A final feature of this profile that stands out is the high probability of risky drinking behavior and the low probability of nondrinking. This profile was labeled *broadly healthy, but high alcohol consumption*.

The second profile also scored favorably on most health behavior dimensions. Fruit and vegetable consumption was high in this profile, and, albeit slightly higher than in the first profile, and levels of smoking were low. Moreover, people with this health behavior profile were highly likely to be nondrinkers. A negative characteristic of this profile was the high likelihood of nonparticipation in sports and of rarely walking for more than 10 minutes continuously. This profile was labeled *low smoking and drinking, healthy nutrition, and physically inactive*.

The third latent health behavior profile identified was characterized by relatively poor scores on most dimensions. People with this profile were relatively likely to smoke and their consumption of fruit and vegetables tended to be low. As with the second profile, they had a high likelihood of nonparticipation in sports and rarely walked for more than 10 minutes. However, they were considerably less likely to report risky drinking behavior and more likely to be nondrinkers than their counterparts in the first health behavior profile. The label *broadly unhealthy, but low alcohol consumption* was therefore assigned to this profile.

As with the *broadly healthy, but high alcohol consumption* profile, people assigned to the final, and least prevalent, profile were relatively likely to have risky drinking behavior. On each of the other dimensions, they scored much more poorly than people with *broadly healthy, but high alcohol consumption* profile, albeit not quite as poorly as the people with the *broadly unhealthy, but low alcohol consumption* profile. This profile was therefore labeled *broadly moderately unhealthy and high alcohol consumption*.

### Early-Life Circumstances and Later-Life Health Behavior Profiles

Table 2 shows the results of the multinomial logistic regression models predicting class membership. Given that, coefficient estimates of multinomial models are difficult to interpret, average marginal effects are presented instead. These can be interpreted as the average discrete change in the predicted probability of having a specific health behavior profile associated with being in the nonreference category of a particular explanatory variable as opposed to the reference category.

**Table 2.** Results of Multinomial Logistic Regression Analyses Predicting Class Membership and Average Marginal Effects.

	HBP 1: Broadly Healthy, High Alcohol Consumption	HBP 2: Low Smoking and Alcohol Consumption, Healthy Nutrition, Physically Inactive	HBP 3: Broadly Unhealthy, Low Alcohol Consumption	HBP 4: Broadly Moderately Unhealthy, High Alcohol Consumption
Parental socioeconomic position				
High	Ref	Ref	Ref	Ref
Intermediate	−.046**	.008	.032*	.006
Low	−.102***	.028*	.063***	.011
Did not grow up with both parents	−.017	.008	.011	−.002
Not born in the UK	−.021	.072***	−.025	−.026
Diagnosed disease at the age of 18 years	−.045*	−.006	.036	.015
Woman	.032**	.107***	−.082***	−.057***
Age	.001	.006***	−.005***	−.002***
Educational attainment				
High	Ref	Ref	Ref	Ref
Intermediate	−.125***	.018	.083***	.025*
Low	−.246***	.043***	.163***	.040***
Marital status				
Married	Ref	Ref	Ref	Ref
Divorced	−.121***	.008	.105***	.008
Widowed	−.085***	.008	.081***	−.004
Never married	−.142***	−.003	.100***	.045*
Number of children				
No children	−.007	.010	.003	−.007
1 child	−.039*	−.002	.027	.015
2 children	Ref	Ref	Ref	Ref
3 children	−.007	−.006	.014	−.001
4 or more children	−.070***	.025	.053**	−.008

*Notes*: Data are from understanding society–The UK Household Longitudinal Study; *n*=4649; Data are weighted; Robust standard errors; Multiple imputation using chained equations used to deal with missing data. * *p* < .05, ** *p* < .01, *** *p* < .001.

The analyses showed that early-life circumstances predicted later-life health behavior profiles. Compared to people whose parents had high socioeconomic position, people whose parents had a lower socioeconomic position were more likely to have the *broadly unhealthy and low alcohol consumption* or the *low smoking and alcohol consumption, healthy nutrition, and physically inactive* health behavior profiles and less likely to have the *broadly healthy, and high alcohol consumption* health behavior profile. These effects were most pronounced for people whose parents had a low socioeconomic, rather than intermediate, position. Moreover, people born outside the UK were more likely than their UK-born counterparts to have a *low smoking and alcohol consumption, healthy nutrition, and physically inactive* health behavior profile, and being diagnosed with a disease at the age of 18 years or younger was associated with a lower likelihood of the *broadly healthy and high alcohol consumption* health behavior profile.

The model moreover showed that women were more likely than men to have the *broadly healthy, but high alcohol consumption* and *low smoking and alcohol consumption, healthy nutrition, and physically inactive* health behavior profiles and less likely to have the *broadly unhealthy, but low alcohol consumption* and *broadly moderately unhealthy and high alcohol consumption* health behavior profiles. Higher age was also associated with a lower likelihood of having the latter two health behavior profiles and a higher likelihood of having the *low smoking and alcohol consumption, healthy nutrition, and physically inactive* health behavior profile.

Compared to their highly educated counterparts, people with lower levels of educational attainment were more likely to have the *broadly unhealthy and low alcohol consumption,* the *low smoking and alcohol consumption, healthy nutrition, and physically inactive*, or the *broadly moderately unhealthy and high alcohol consumption* health behavior profiles and less likely to have the *broadly healthy and high alcohol consumption* health behavior profile. These effects were most pronounced for people with low, rather than intermediate, levels of education. Married people were more likely to have the *broadly healthy and high alcohol consumption* health behavior profile and less likely to have the *broadly unhealthy and low alcohol consumption* health behavior profile than divorced, widowed, and never married people. Few health behavior differences by number of children were found.

### Allostatic Load

Table 3 shows the results of the linear regression models predicting allostatic load. The first model showed that allostatic load was significantly higher for people whose parents had an intermediate or low socioeconomic position than for people with high socioeconomic parents. The model further showed that higher age, male gender, lower educational attainment, being divorced or married as opposed to married, and having one or four or more children as opposed to two children were associated with higher allostatic load.

**Table 3.** Results of Linear Regression Models of Allostatic Load.

	Model 1		Model 2	
	B	(*SE*)	B	(*SE*)
Early-life characteristics				
Parental socioeconomic position				
High	Ref		Ref	
Intermediate	.192*	(.087)	.160	(.085)
Low	.401***	(.091)	.330***	(.090)
Did not grow up with both parents	.169	(.097)	.156	(.096)
Not born in the UK	.083	(.141)	.068	(.139)
Diagnosed disease at the age of 18	.180	(.160)	.150	(.157)
Health behavior profiles (HBPs)				
HBP 1: Broadly healthy and high alcohol consumption			Ref	
HBP 2: Low smoking and alcohol consumption, healthy nutrition, and physically inactive			.629***	(.054)
HBP 3: Broadly unhealthy and low alcohol consumption			.791***	(.077)
HBP 4: Broadly moderately unhealthy and high alcohol consumption			.377***	(.068)
Woman	−.149*	(.071)	−.129	(.071)
Age	.029***	(.004)	.030***	(.004)
Educational attainment				
High	Ref		Ref	
Intermediate	.356***	(.099)	.269**	(.098)
Low	.734***	(.094)	.561***	(.094)
Marital status				
Married	Ref		Ref	
Divorced	.336***	(.097)	.243*	(.096)
Widowed	.346**	(.121)	.278*	(.119)
Never married	−.000	(.151)	−.099	(.149)
Number of children				
No children	.156	(.109)	.149	(.107)
1 child	.230*	(.117)	.204	(.114)
2 children	Ref		Ref	
3 children	.171	(.092)	.164	(.091)
4 or more children	.427***	(.123)	.373**	(.122)
Constant	.449	(.288)	.210	(.285)

*Notes*: HBP = Health behavior profiles, Data are from Understanding Society–The UK Household Longitudinal Study; *n* = 4649; Data are weighted; Robust standard errors; Multiple imputation using chained equations used to deal with missing data. * *p* < .05, ** *p* < .01, *** *p* < .001.

In the second model, the health behavior profiles identified in the LCA were added. People with a *broadly healthy and high alcohol consumption* had significantly lower allostatic load than people in any of the other health behavior profiles. Allostatic load was Furthermore significantly higher for people with a *low smoking and alcohol consumption, healthy nutrition ,and physically inactive* (Δ*b* = .251, *p* < .01) or *broadly unhealthy and low alcohol consumption* (Δ*b* = .414, *p* < .001) health behavior profile than for people with a *broadly moderately unhealthy and high alcohol consumption* profile.

The addition of early-life characteristics of the health behavior profiles to the model attenuated the effects of parental socioeconomic position. As shown in Table 4, the indirect effects of low and intermediate versus high parental socioeconomic position via the health behavior profiles were both statistically significant. The indirect effect via health behavior profiles accounted for 17.9% of the total effect of low as opposed to high parental socioeconomic position and for 17.0% of the total effect of intermediate as opposed to high parental socioeconomic position as estimated in Model 1 in Table 3. Approximately, 70% of the indirect effects of parental socioeconomic position via health behavior profiles were driven by the greater disposition of people with low and intermediate socioeconomic position parents than their counterparts with high socioeconomic position parents to have a *broadly unhealthy and low alcohol consumption* as opposed to a *broadly healthy and high alcohol consumption* health behavior profile.

**Table 4.** Decomposition of Coefficients of Parental Socio-Economic Position.

	Low versus high					Intermediate versus high				
	B	(*SE*)	*p*	Share total effect (%)	Share indirect effect (%)	B	(*SE*)	*p*	Share total effect (%)	Share indirect effect (%)
Δb Model 2 – Model 1	.072	(.012)	<.001	17.9	100.0	.032	(.011)	<.01	17.0	100.0
Components of difference										
Health behavior profiles (HBPs)										
HBP 1: Broadly healthy, and high alcohol consumption	Ref					Ref				
HBP 2: Low smoking and alcohol consumption, healthy nutrition, and physically inactive	.018	(.009)	.052	4.4	24.7	.006	(.008)	.302	3.2	19.1
HBP 3: Broadly unhealthy and low alcohol consumption	.050	(.012)	<.001	12.4	69.2	.023	(.010)	<.05	12.2	71.8
HBP 4: Broadly moderately unhealthy and high alcohol consumption	.004	(.004)	.245	1.1	6.1	.003	(.004)	.316	1.5	9.1

*Notes*. HBP = Health behavior profiles, Data are from Understanding Society–The UK Household Longitudinal Study; *n*=4649; Data are weighted; Bootstrapped standard errors; Multiple imputation using chained equations used to deal with missing data.

## Discussion

It is well established that growing up under disadvantageous circumstances, such as in poor financial conditions, is associated with poor later-life health. The current study assessed a potential pathway from early-life circumstances to later-life health via health behavior. Latent class analysis (LCA) was used to distinguish particular health behavior profiles that vary on multiple behavioral dimensions–smoking, nutrition, alcohol consumption, and physical activity (SNAP) – among people aged 50–80 years in Great Britain. Multinomial logistic regression analyses then shed light on how disadvantageous early-life circumstances are associated with having particular health behavior profiles. Finally, the current study assessed to what extent the effects of disadvantageous early-life circumstances on allostatic load–an objective measure of the physiological wear and tear of the body–were mediated by the health behavior profiles identified in the latent class analysis.

Four distinct health behavior profiles were identified among the older British population: (1) *broadly healthy and high alcohol consumption*, (2) *low smoking and alcohol consumption*, *healthy nutrition, and physically inactive*, (3) *broadly unhealthy and low alcohol consumption,* and (4) *broadly moderately unhealthy and high alcohol consumption*. Marked differences in health behavior profiles were found by parental socioeconomic position, but few systematic differences were found by the other early-life characteristics considered: growing up in a single-parent family, not being born in the UK, and being diagnosed with a health condition at the age of 18 years or younger.

The analyses furthermore showed that people who grew up in a high socioeconomic position family had lower later-life allostatic load than their counterparts who grew up in families with a lower socioeconomic position, and that these differences could to a substantial extent be attributed to differences in health behavior profiles. The lion’s share of the indirect effects of parental socioeconomic position via health behavior profiles was due to people with low and intermediate socioeconomic position parents’ high likelihood to have the health behavior profile associated with the highest allostatic load in later life (*broadly unhealthy and low alcohol consumption*) and their low likelihood to have the health profile with the lowest allostatic load (*broadly healthy and high alcohol consumption*).

The current study extended earlier work by life course epidemiologists on later-life health by assessing a chain risk between early-life circumstances and later-life health via health behavior, in a way that acknowledged that health behavior is a multidimensional concept. Much earlier work explored chains of risks via socioeconomic circumstances in adulthood (e.g., Luo & Waite, 2005; Surachman et al., 2019), and the few studies that explored chains of risk via health behavior have tended to combine multiple aspects of health behavior into an index (Barboza Solís et al., 2016). Combining multiple aspects of health behavior into an index is, however, at odds with the increasingly dominant view that health behavior is a multidimensional concept. One of the reasons for this is that various forms of health behavior are only weakly associated (Newsom et al., 2005). In line with this argument, a reliability analysis indicated that the six health behavior items considered in the current study did not form an internally consistent scale together (*α* = .35). Moreover, the effects of various forms of health behavior may offset or compound each other (e.g., Shaw & Agahi, 2012). The results presented here are a case in point. The two most prevalent health behavior profiles (*broadly healthy and high alcohol consumption* and *low smoking and alcohol consumption*, *healthy nutrition, and physically inactive*) were both characterized by favorable scores on three of the four SNAP dimensions of health behavior. This implies that people with either of these two health behavior profiles would have roughly similar scores on an index in which multiple aspects of health behavior are simply summed. Nevertheless, the analyses indicated that early-life antecedents as well as the associations with allostatic load differed notably between these two behavior profiles. This underlines the importance of acknowledging the multidimensional nature of health behavior when investigating the mediating pathways of early-life characteristics on later-life health via health behavior.

The main limitation of the current study is that the analyses are cross sectional. Allostatic load was regressed on health behavior profiles, but it is not implausible that health status may also predispose people to a specific health behavior profile. In particular, having a *low smoking and alcohol consumption, healthy nutrition, and physically inactive* health behavior profile may be driven by health problems. Health problems may, for instance, be a barrier for physical activity (Xiang, 2016) and a reason for medical practitioners to urge the person with health problems to abstain from alcohol and smoking. However, earlier research showed that only few persons diagnosed with a new chronic condition adopt healthier behaviors (Newsom et al., 2012). Moreover, this study did not show an association between being diagnosed with a disease when growing up and having this particular health behavior profile. Nevertheless, analyses of birth cohort data with frequently repeated measures of health status and health behavior are needed to shed light on the ways in which health behavior profiles and health mutually shape each other.

A second notable limitation is that the information on early-life circumstances used in the current study was collected retrospectively, as were some important control variables. Retrospectively collected information may be of lower quality than prospectively collected information (cf. Sironi et al., 2020) because it may be prone to recall bias. In a recent study, in which prospective and prospective data on British people in their 50s were compared, no systematic differences in the distribution of important early-life characteristics (socioeconomic position of father when growing up and parental separation between birth and age of 16 years) were found, but the authors noted that retrospectively collected early-life characteristics were slightly, but statistically significantly, more strongly associated with later-life health than were prospectively collected early-life characteristics (Jivraj et al., 2020). This suggests that the magnitude of the impact of parental socioeconomic position on later-life allostatic load may be overestimated in the current study.

Recall bias may also explain the considerably higher levels of reported childlessness among men than among women in the sample analyzed here. Rendall et al. (1999) analyzed fertility histories in the British Household Panel Study and the Panel Study on Income Dynamics concluded that between one-third and half of the men’s nonmarital births and births within previous marriages were missed when information on fertility histories was collected retrospectively. Men’s higher levels of reported childlessness in the current study may, however, to some extent also capture the fact that, in couples, men tend to be older than women. Female partners of male respondents are thus typically born later than female respondents. This is important because the median of birth year of female respondents was 1948, and women born in the second half of 1940s form a cohort with a considerably lower prevalence of childlessness than subsequent cohorts of women (Berrington, 2017).

It should also be considered that whereas multinomial logistic regression was used to predict health behavior profiles, the indirect effects of parental socioeconomic position were calculated based on linear probability estimates of class membership (cf. Preacher & Hayes, 2008). However, these linear probability estimates (see Supplemental Appendix C) were very similar to average marginal effects from the multinomial logistic regression model of class membership presented in Table 2.

Older adults’ health behavior patterns tend to be highly stable over time (Burgard et al., 2020), and even after the development of health conditions, people find it difficult to adopt a healthier lifestyle (Newsom et al., 2012). This suggests that efforts to disrupt the chain of risk from early-life socioeconomic disadvantage to poorer later-life health via health behavior may be most promising if they focus on early phases in the life course. Recent reviews suggested that interventions targeting disadvantaged children may yield modest improvements in health behavior among this group (Craike et al., 2018; Wijtzes et al., 2017). However, the body of evidence on which interventions can be based is still very limited considering the need to battle the suboptimal health behavior choices that follow from growing up under disadvantageous circumstances. Wijtzes et al. (2017) therefore recently called for increasing scholarly attention for the long-term effectiveness of interventions aiming to improve health behavior among children growing up under disadvantageous circumstances. The current study’s finding of an important pathway from early-life socioeconomic disadvantage to poorer later-life health via a disposition for suboptimal health behavior underlines the urgency of this call.

## Supplemental Material

sj-pdf-1-jah-10.1177_0898264320981233 – Supplemental Material for Early-Life Circumstances, Health Behavior Profiles, and Later-Life Health in Great Britain Click here for additional data file. Supplemental Material, sj-pdf-1-jah-10.1177_0898264320981233 for Early-Life Circumstances, Health Behavior Profiles, and Later-Life Health in Great Britain by Thijs van den Broek in Journal of Aging and Health

## References

[bibr1-0898264320981233] Abel T. (2008). Cultural capital and social inequality in health. Journal of Epidemiology and Community Health, 62(7), e13. doi:10.1136/jech.2007.066159 18572429

[bibr2-0898264320981233] Akaike H. (1974). A new look at the statistical model identification. IEEE Transactions on Automatic Control, 19(6), 716-723. doi:10.1109/TAC.1974.1100705

[bibr3-0898264320981233] Bakk Z. Vermunt J. K. (2016). Robustness of stepwise latent class modeling with continuous distal outcomes. Structural Equation Modeling: A Multidisciplinary Journal, 23(1), 20-31. doi:10.1080/10705511.2014.955104

[bibr4-0898264320981233] Barboza Solís C. Fantin R. Castagné R. Lang T. Delpierre C. Kelly-Irving M. (2016). Mediating pathways between parental socio-economic position and allostatic load in mid-life: Findings from the 1958 British birth cohort. Social Science & Medicine, 165, 19-27. doi:10.1016/j.socscimed.2016.07.031 27485729

[bibr5-0898264320981233] Benzeval M. Davillas A. Kumari M. Lynn P. (2014). Understanding society: The UK household longitudinal study biomarker user guide and glossary. Colchester: Institute for Social and Economic Research.

[bibr6-0898264320981233] Berrington A. (2017). Childlessness in the UK. In Kreyenfeld M. Konietzka D. (Eds.), Childlessness in Europe: Contexts, causes, and consequences (pp. 57-76). Springer. doi:10.1007/978-3-319-44667-7_3

[bibr7-0898264320981233] Bolck A. Croon M. Hagenaars J. (2004). Estimating latent structure models with categorical variables: One-step versus three-step estimators. Political Analysis, 12(1), 3-27. doi:10.1093/pan/mph001

[bibr8-0898264320981233] Brand J. Buuren S. Cessie S. Hout W. (2019). Combining multiple imputation and bootstrap in the analysis of cost‐effectiveness trial data. Statistics in Medicine, 38(2), 210-220. doi:10.1002/sim.7956 30207407 PMC6585698

[bibr9-0898264320981233] Burgard S. A. Lin K. Y. P. Segal B. D. Elliott M. R. Seelye S. (2020). Stability and change in health behavior profiles of U.S. adults. The Journals of Gerontology: Series B, 75(3), 674-683. doi:10.1093/geronb/gby088 PMC7328035 32059056

[bibr10-0898264320981233] Chinn D. J. White M. Harland J. Drinkwater C. Raybould S. (1999). Barriers to physical activity and socioeconomic position: Implications for health promotion. Journal of Epidemiology and Community Health, 53(3), 191-192. doi:10.1136/jech.53.3.191 10396499 PMC1756843

[bibr11-0898264320981233] Cockerham W. C. (2005). Health lifestyle theory and the convergence of agency and structure. Journal of Health and Social Behavior, 46(1), 51-67. doi:10.1177/002214650504600105 15869120

[bibr12-0898264320981233] Craike M. Wiesner G. Hilland T. A. Bengoechea E. G. (2018). Interventions to improve physical activity among socioeconomically disadvantaged groups: An umbrella review. International Journal of Behavioral Nutrition and Physical Activity, 15(1), 43. doi:10.1186/s12966-018-0676-2 PMC5952843 29764488

[bibr13-0898264320981233] Danese A. McEwen B. S. (2012). Adverse childhood experiences, allostasis, allostatic load, and age-related disease. Physiology & Behavior, 106(1), 29-39. doi:10.1016/j.physbeh.2011.08.019 21888923

[bibr14-0898264320981233] Dempster A. P. Laird N. M. Rubin D. B. (1977). Maximum likelihood from incomplete data via the EM algorithm. Journal of the Royal Statistical Society: Series B (Methodological), 39(1), 1-22. doi:10.2307/2984875

[bibr15-0898264320981233] Department of Health and Social Care (2016). UK chief medical officers’ low risk drinking guidelines. Department of Health and Social Care.

[bibr16-0898264320981233] Drewnowski A. (2013). The economics of food choice behavior: Why poverty and obesity are linked. In Drewnowski A. Rolls B. J. (Eds.), Obesity treatment and prevention: New directions (pp. 95-112). Karger. doi:10.1159/000341303 23128769

[bibr17-0898264320981233] Dronkers J. Härkönen J. (2008). The intergenerational transmission of divorce in cross-national perspective: Results from the fertility and family surveys. Population Studies, 62(3), 273-288. doi:10.1080/00324720802320475 18937142

[bibr18-0898264320981233] Dziak J. J. Coffman D. L. Lanza S. T. Li R. Jermiin L. S. (2020). Sensitivity and specificity of information criteria. Briefings in Bioinformatics, 21(2), 553-565. doi:10.1093/bib/bbz016 30895308 PMC7299313

[bibr19-0898264320981233] Gilman S. E. Kawachi I. Fitzmaurice G. M. Buka S. L. (2003). Family disruption in childhood and risk of adult depression. American Journal of Psychiatry, 160(5), 939-946. doi:10.1176/appi.ajp.160.5.939 12727699

[bibr20-0898264320981233] Griffin B. Sherman K. A. Jones M. Bayl-Smith P. (2014). The clustering of health behaviours in older Australians and its association with physical and psychological status, and sociodemographic indicators. Annals of Behavioral Medicine, 48(2), 205-214. doi:10.1007/s12160-014-9589-8 24500081 PMC4212155

[bibr21-0898264320981233] Gruenewald T. L. Karlamangla A. S. Hu P. Stein-Merkin S. Crandall C. Koretz B. Seeman T. E. (2012). History of socioeconomic disadvantage and allostatic load in later life. Social Science & Medicine, 74(1), 75-83. doi:10.1016/j.socscimed.2011.09.037 22115943 PMC3264490

[bibr22-0898264320981233] Grundy E. Read S. (2015). Pathways from fertility history to later life health: Results from analyses of the English longitudinal study of ageing. Demographic Research, 32(1), 107-146. doi:10.4054/DemRes.2015.32.4

[bibr23-0898264320981233] Grundy E. Van den Broek T. Keenan K. (2019). Number of children, partnership status, and later-life depression in eastern and western Europe. The Journals of Gerontology: Series B, 74(2), 353-363. doi:10.1093/geronb/gbx050 PMC6327656 28472400

[bibr24-0898264320981233] Hofmann W. Friese M. Wiers R. W. (2008). Impulsive versus reflective influences on health behavior: A theoretical framework and empirical review. Health Psychology Review, 2(2), 111-137. doi:10.1080/17437190802617668

[bibr25-0898264320981233] Högnäs R. S. Roelfs D. J. Shor E. Moore C. Reece T. (2017). J-curve? A meta-analysis and meta-regression of parity and parental mortality. Population Research and Policy Review, 36(2), 273-308. doi:10.1007/s11113-016-9421-1

[bibr26-0898264320981233] Hughes A. Kumari M. (2017). Unemployment, underweight, and obesity: Findings from understanding society (UKHLS). Preventive Medicine, 97, 19-25. doi:10.1016/j.ypmed.2016.12.045 28034731

[bibr27-0898264320981233] Jenkins S. P. Siedler T. (2007). The intergenerational transmission of poverty in industrialized countries. CPRC working paper 75. Colchester: Institute for Social and Economic Research.

[bibr28-0898264320981233] Jivraj S. Goodman A. Ploubidis G. B. de Oliveira C. (2020). Testing comparability between retrospective life history data and prospective birth cohort study data. The Journals of Gerontology: Series B, 75(1), 207-217. doi:10.1093/geronb/gbx042 PMC6909437 28444303

[bibr29-0898264320981233] Johnson S. C. Cavallaro F. L. Leon D. A. (2017). A systematic review of allostatic load in relation to socioeconomic position: Poor fidelity and major inconsistencies in biomarkers employed. Social Science & Medicine, 192, 66-73. doi:10.1016/j.socscimed.2017.09.025 28963986

[bibr30-0898264320981233] Kuh D. Ben-Shlomo Y. Lynch J. Hallqvist J. Power C. (2003). Life course epidemiology. Journal of Epidemiology and Community Health, 57(10), 778-783. doi:10.1136/jech.57.10.778 14573579 PMC1732305

[bibr31-0898264320981233] Kumari M. Head J. Bartley M. Stansfeld S. Kivimaki M. (2013). Maternal separation in childhood and diurnal cortisol patterns in mid-life: Findings from the whitehall II study. Psychological Medicine, 43(3), 633-643. doi:10.1017/S0033291712001353 22785027

[bibr32-0898264320981233] Latham-Mintus K. Aman K. M. (2019). Childhood disadvantage, psychosocial resiliency, and later life functioning: Linking early-life circumstances to recovery from mobility limitation. Journal of Aging and Health, 31(3), 463-483. doi:10.1177/0898264317733861 29254410

[bibr33-0898264320981233] Little R. J. A. Rubin D. B. (1989). The analysis of social science data with missing values. Sociological Methods & Research, 18(2–3), 292-326. doi:10.1177/0049124189018002004

[bibr34-0898264320981233] Lorenz F. O. Wickrama K. A. S. Conger R. D. Elder G. H. (2006). The Short-term and decade-long effects of divorce on women's midlife health. Journal of Health and Social Behavior, 47(2), 111-125. doi:10.1177/002214650604700202 16821506

[bibr35-0898264320981233] Luo Y. Waite L. J. (2005). The impact of childhood and adult SES on physical, mental, and cognitive well-being in later life. The Journals of Gerontology: Series B, 60(2), S93-S101. doi:10.1093/geronb/60.2.S93 PMC2505177 15746030

[bibr36-0898264320981233] McCutcheon A. L. (1987). Latent class analysis. Thousand Oaks, CA: Sage.

[bibr37-0898264320981233] McEwen B. S. (1998). Stress, adaptation, and disease: Allostasis and allostatic load. Annals of the New York Academy of Sciences, 840(1), 33-44. doi:10.1111/j.1749-6632.1998.tb09546.x 9629234

[bibr38-0898264320981233] McEwen B. S. Stellar E. (1993). Stress and the individual. mechanisms leading to disease. Archives of Internal Medicine, 153(18), 2093-2101. doi:10.1001/archinte.1993.00410180039004 8379800

[bibr39-0898264320981233] Newsom J. T. Huguet N. McCarthy M. J. Ramage-Morin P. Kaplan M. S. Bernier J. Oderkirk B. H.. (2012) Health behavior change following chronic illness in middle and later life. The Journals of Gerontology: Series B, 67B(3), 279-288. doi:10.1093/geronb/gbr103 PMC3325087 21983040

[bibr40-0898264320981233] Newsom J. T. McFarland B. H. Kaplan M. S. Huguet N. Zani B. (2005). The health consciousness myth: Implications of the near independence of major health behaviors in the north american population. Social Science & Medicine, 60(2), 433-437. doi:10.1016/j.socscimed.2004.05.015 15522497

[bibr41-0898264320981233] Pakpahan E. Hoffmann R. Kröger H. (2017). The long arm of childhood circumstances on health in old age: Evidence from SHARELIFE. Advances in Life Course Research, 31(Supplement C), 1-10. doi:10.1016/j.alcr.2016.10.003

[bibr42-0898264320981233] Peterson J. L. Zill N. (1986). Marital disruption, parent-child relationships, and behavior problems in children. Journal of Marriage and the Family, 48(2), 295-307. doi:10.2307/352397

[bibr43-0898264320981233] Präg P. Richards L. (2019). Intergenerational social mobility and allostatic load in Great Britain. Journal of Epidemiology and Community Health, 73, 100-105. doi:10.1136/jech-2017-210171 30385515

[bibr44-0898264320981233] Preacher K. J. Hayes A. F. (2008). Asymptotic and resampling strategies for assessing and comparing indirect effects in multiple mediator models. Behavior Research Methods, 40(3), 879-891. doi:10.3758/BRM.40.3.879 18697684

[bibr45-0898264320981233] Quinlivan J. A. Tan L. H. Steele A. Black K. (2004). Impact of demographic factors, early family relationships and depressive symptomatology in teenage pregnancy. Australian & New Zealand Journal of Psychiatry, 38(4), 197-203. doi:10.1080/j.1440-1614.2004.01336.x 15038797

[bibr46-0898264320981233] Raphael D. (2011). Poverty in childhood and adverse health outcomes in adulthood. Maturitas, 69(1), 22-26. doi:10.1016/j.maturitas.2011.02.011 21398059

[bibr47-0898264320981233] Rendall M. S. Clarke L. Peters H. E. Ranjit N. Verropoulou G. (1999). Incomplete reporting of men’s fertility in the United States and Britain: A research note. Demography, 36(1), 135-144. doi:10.2307/2648139 10036598

[bibr48-0898264320981233] Robert S. House J. S. (1996). SES Differentials in health by age and alternative indicators of SES. Journal of Aging and Health, 8(3), 359-388. doi:10.1177/089826439600800304 10165980

[bibr49-0898264320981233] Rote S. (2017). Marital disruption and allostatic load in late life. Journal of Aging and Health, 29(4), 688-707. doi:10.1177/0898264316641084 27079918

[bibr50-0898264320981233] Saint Onge J. M. Krueger P. M. (2017). Health lifestyle behaviors among U.S. adults. SSM - Population Health, 3, 89-98. doi:10.1016/j.ssmph.2016.12.009 28785602 PMC5544030

[bibr51-0898264320981233] Schwarz G. (1978). Estimating the dimension of a model. The Annals of Statistics, 6(2), 461-464. doi:10.1214/aos/1176344136

[bibr52-0898264320981233] Seeman T. E. McEwen B. S. Rowe J. W. Singer B. H. (2001). Allostatic load as a marker of cumulative biological risk: MacArthur studies of successful aging. Proceedings of the National Academy of Sciences, 98(8), 4770-4775. doi:10.1073/pnas.081072698 PMC31909 11287659

[bibr53-0898264320981233] Shaw B. A. Agahi N. (2012). A prospective cohort study of health behavior profiles after age 50 and mortality risk. BMC Public Health, 12(1), 803. doi:10.1186/1471-2458-12-803 22989155 PMC3503621

[bibr54-0898264320981233] Sironi M. Ploubidis G. B. Grundy E. M. (2020). Fertility history and biomarkers using prospective data: Evidence from the 1958 national child development study. Demography, 57(2), 529-558. doi:10.1007/s13524-020-00855-x 32133595 PMC7162827

[bibr55-0898264320981233] Surachman A. Wardecker B. Chow S.-M. Almeida D. (2019). Life course socioeconomic status, daily stressors, and daily well-being: Examining chain of risk models. The Journals of Gerontology: Series B, 74(1), 126-135. doi:10.1093/geronb/gby014 PMC6294233 29669043

[bibr56-0898264320981233] Tein J.-Y. Coxe S. Cham H. (2013). Statistical power to detect the correct number of classes in latent profile analysis. Structural Equation Modeling: A Multidisciplinary Journal, 20(4), 640-657. doi:10.1080/10705511.2013.824781 24489457 PMC3904803

[bibr57-0898264320981233] University of Essex, Institute for Social and Economic Research, NatCen Social Research, & Kantar Public (2018). Understanding society: Waves 1-8, 2009-2017 and harmonised BHPS: Waves 1-18, 1991-2009 (11th ed.). University of Essex. doi:10.5255/UKDA-SN-6614-12

[bibr58-0898264320981233] University of Essex, Institute for Social and Economic Research, NatCen Social Research, & Kantar Public (2019). Understanding society: Waves 2 and 3 nurse health assessment, 2010- 2012 (3rd ed). University of Essex. doi:10.5255/UKDA-SN-7251-3

[bibr59-0898264320981233] Wardle J. Steptoe A. (2003). Socioeconomic differences in attitudes and beliefs about healthy lifestyles. Journal of Epidemiology and Community Health, 57(6), 440-443. doi:10.1136/jech.57.6.440 12775791 PMC1732468

[bibr60-0898264320981233] Whitley E. Benzeval M. Popham F. (2018). Associations of successful aging with socioeconomic position across the life-course: The west of scotland twenty-07 prospective cohort study. Journal of Aging and Health, 30(1), 52-74. doi:10.1177/0898264316665208 27581420 PMC5714158

[bibr61-0898264320981233] Wijtzes A. I. Van de Gaar V. M. Van Grieken A. De Kroon M. L. A. Mackenbach J. P. Van Lenthe F. J., &Raat W. (2017). Effectiveness of interventions to improve lifestyle behaviors among socially disadvantaged children in Europe. European Journal of Public Health, 27(2), 240-247. doi:10.1093/eurpub/ckw136 28375430

[bibr62-0898264320981233] Woodward L. Fergusson D. M. Horwood L. J. (2004). Risk factors and life processes associated with teenage pregnancy: Results of a prospective study from birth to 20 years. Journal of Marriage and Family, 63(4), 1170-1184. doi:10.1111/j.1741-3737.2001.01170.x

[bibr63-0898264320981233] Xiang X.. (2016). Chronic disease diagnosis as a teachable moment for health behavior changes among middle-aged and older adults. Journal of Aging and Health, 28(6), 995-1015. doi:10.1177/0898264315614573 26634998

[bibr64-0898264320981233] Zaninotto P. Head J. Steptoe A. (2020). Behavioural risk factors and healthy life expectancy: Evidence from two longitudinal studies of ageing in England and the US. Scientific Reports, 10(1), 6955. doi:10.1038/s41598-020-63843-6 32332825 PMC7181761

