# Supplementary material for: Early-Life Circumstances, Health Behavior Profiles, and Later-Life Health in Great Britain
Source: J Aging Health. 2020 Dec 19;33(5-6):317–30. doi: 10.1177/0898264320981233 (PMC8120632; doi:10.1177/0898264320981233)
Supplement: sj-pdf-1-jah-10.1177_0898264320981233 – Supplemental Material for Early-Life Circumstances, Health Behavior Profiles, and Later-Life Health in Great Britain [file sj-pdf-1-jah-10.1177_0898264320981233.pdf]

Appendix A. Operationalization of allostatic load

Marker	Biological system	Flag
Systolic blood pressure	Cardiovascular	Top quartile in sex-specific weighted distribution
Diastolic blood pressure	Cardiovascular	Top quartile in sex-specific weighted distribution
Resting heart rate	Cardiovascular	Top quartile in sex-specific weighted distribution
Waist-to-height ratio	Metabolic	Top quartile in sex-specific weighted distribution
Total cholesterol to HDL ratio	Metabolic	Top quartile in sex-specific weighted distribution
HDL cholesterol	Metabolic	Bottom quartile in sex-specific weighted distribution
Triglycerides	Metabolic	Top quartile in sex-specific weighted distribution
Glycated Haemoglobin	Metabolic	Top quartile in sex-specific weighted distribution
Insulin-like growth factor-1	Metabolic	Bottom quartile in sex-specific weighted distribution
C-reactive protein	Immune response	Top quartile in sex-specific weighted distribution
Fibrinogen	Immune response	Top quartile in sex-specific weighted distribution
Creatinine clearance rate	Kidney / liver function	Top quartile in sex-specific weighted distribution
Dihydroepiandrosterone sulphate	HPA-Axis	Bottom quartile in sex-specific weighted distribution

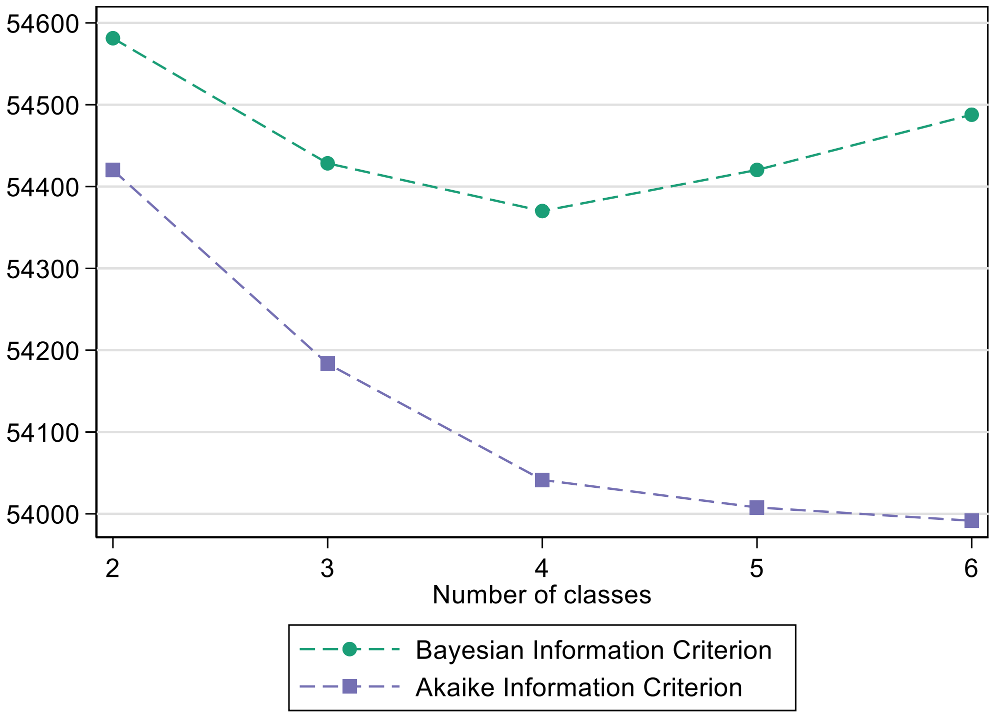

Appendix B. Fit statistics for latent class analysis by number of classes

Appendix C. Results of linear probability models of class membership.

	HBP1: Broadly healthy, high alcohol consumption	HBP2: Low smoking and alcohol consumption, healthy nutrition, physically inactive	HBP3: Broadly unhealthy, low alcohol consumption	HBP4: Broadly moderately unhealthy, high alcohol consumption
Parental socio-economic position:				
High	Ref.	Ref.	Ref.	Ref.
Intermediate	-0.047**	0.010	0.029*	0.008
Low	-0.102***	0.028*	0.063***	0.012
Did not grow up with both parents	-0.017	0.008	0.011	-0.003
Not born in UK	-0.020	0.067***	-0.022	-0.026
Diagnosed disease at age 18	-0.045*	-0.006	0.035	0.015
Woman	0.033**	0.108***	-0.083***	-0.057***
Age	0.001	0.007***	-0.0052***	-0.002***
Educational Attainment:				
High	Ref.	Ref.	Ref.	Ref.
Intermediate	-0.129***	0.016	0.085***	0.029*
Low	-0.250***	0.041***	0.166***	0.043***
Marital status:				
Married	Ref.	Ref.	Ref.	Ref.
Divorced	-0.121***	0.004	0.111***	0.007
Widowed	-0.087***	0.015	0.075***	-0.003
Never married	-0.147***	-0.004	0.106***	0.045*
Number of children:				
No children	-0.007	0.011	0.003	-0.007
1 child	-0.041*	-0.002	0.028	0.015
2 children	Ref.	Ref.	Ref.	Ref.
3 children	-0.008	-0.006	0.015	-0.001
4 or more children	-0.069***	0.025	0.052**	-0.009
Constant	0.595***	-0.272***	0.375***	0.302***

Notes: Data are from Understanding Society – The UK Household Longitudinal Study; n=4,649; Data are weighted; Cluster robust standard errors; Multiple imputation using chained equations used to deal with missing data.
** p < .05, ** p < .01, *** p < .001*
